# Disruption of electrophysiological rhythms and memory impairment in an Alzheimer’s transgenic rat model

**DOI:** 10.1186/s13195-025-01841-4

**Published:** 2025-09-01

**Authors:** Xiaoxiao Tao, Udaya Kumar, Miaomiao Wang, Kapil Manglani, Cansheng Zhu, Mychica R. Jones, Alexander Bombino, Anatol Bragin, Gregory Cole, Keith Vossel, Jerome Engel Jr., Sally A. Frautschy, Lin Li

**Affiliations:** 1 Department of Biomedical Engineering, University of North Texas, Denton, TX 76207 USA; 2 Department of Neurology, University of California, Los Angeles, CA 90095 USA; 3 Geriatric Research, Education and Clinical Center, Veterans Greater Los Angeles Healthcare System, Los Angeles, CA 90073 USA; 4 Geffen School of Medicine, Department of Medicine, University of California, Los Angeles, CA 90095 USA; 5 Brain Research Institute, University of California, Los Angeles, CA 90095 USA; 6 Department of Neurobiology, David Geffen School of Medicine at UCLA, Los Angeles, CA 90095 USA; 7 Department of Psychiatry and Biobehavioral Sciences, David Geffen School of Medicine at UCLA, Los Angeles, CA 90095 USA

**Keywords:** Alzheimer’s disease, High-frequency oscillations, Interictal epileptic spikes, Aβ-immunoreactive plaques, pTau217 pathology

## Abstract

**Background:**

Alzheimer’s disease (AD) is one of the most prevalent causes of dementia, characterized by progressive memory loss and cognitive decline. Abnormal electrophysiological patterns, especially interictal epileptiform discharges (IEDs) and high-frequency oscillations (HFOs), have been observed in mouse models of AD and are suggested to contribute to cognitive dysfunction. However, comprehensive evaluations of IEDs across different brain regions are limited, and their impact on cognitive performance and neuropathology remains unclear, particularly in more complex AD models with relevant comorbidities. To address this gap, our study aims to clarify how IEDs and HFOs contribute to cognitive decline and neuropathology in AD, potentially informing the development of new biomarkers for early detection.

**Methods:**

We investigate these effects in an AD (PS1/APP) rat model (FAD+) with coexisting hypertension-associated small vessel disease (SVD), as well as in their transgene-negative littermates (FAD-). We conducted behavioral experiments at 6, 8, and 11 months of animal age, alongside neural signal recordings at 8 and 11 months. AD pathology (neuritic plaques and hyperphosphorylated tau) and novel biomarkers (14-3-3γ) or biomarkers common to both disorders (neuropeptide Y, astrocyte and microglia) were evaluated at the end of the experiment.

**Results:**

Seizures were observed in three out of 14 FAD + rats. IED rates were significantly greater in FAD + rats compared to FAD- at all tested periods, correlating with changes in neuropathological biomarkers. Furthermore, coupling strength between IEDs and HFOs was significantly elevated in FAD + rats, especially during the later stages of disease progression. In addition, FAD + rats exhibited deficits in both learning and recall abilities at both ages, which correlated most strongly with increased IED–HFO coupling strength. No such correlation was observed in the FAD- group.

**Conclusion:**

Our findings suggest that pathological synchronization between IEDs and HFOs in the hippocampus, along with neuropathological changes in both the hippocampus and entorhinal cortex, may contribute to memory dysfunction in AD, highlighting a potential mechanistic link between epileptiform activity, AD biomarker changes, and cognitive decline.

**Supplementary Information:**

The online version contains supplementary material available at 10.1186/s13195-025-01841-4.

## Introduction

Alzheimer’s disease (AD) is one of the most serious brain disorders and a prevalent form of dementia that progressively impairs memory and cognitive functions [1, 2]. Growing evidence suggests that abnormal electrical activity in the prodromal period may be an important contributor to AD onset and progression [3–6]. Reported electrical disturbances include disruptions in neural oscillations, such as high-frequency oscillations (HFOs), which can include pathological forms (pHFOs, e.g., fast ripples), as well as interictal epileptiform discharges (IEDs) and other irregular brain activity. These abnormal patterns may serve as biomarkers of cognitive deficits [7, 8]. However, given the paucity of invasive electrophysiological measurements with depth electrode implantation in patients with AD, animal models are critical for dissecting the mechanisms underlying these abnormal signals, which remain incompletely understood [9, 10]. Despite progress, knowledge gaps persist. For instance, comprehensive assessments of region-specific changes are lacking, and associations between electrical disturbances and cognitive decline require further exploration in models with greater cognitive complexity than mice. Moreover, the role of comorbidities such as small vessel disease (SVD), which exacerbates the link between dementia and epilepsy, warrants further investigation [11–13]. Addressing these knowledge gaps could enhance our understanding of the mechanisms underlying AD and contribute to the development of targeted therapeutic strategies.

Previous studies have demonstrated that IEDs and pHFOs represent pathological neural activities closely associated with impaired learning and memory performance [4, 7, 14–16]. HFOs are generally defined as oscillations ranging from 80 to 500 Hz [17]. Based on their frequency range, two types have been described: physiological ripples (80–200 Hz) and pathological fast ripples (200–500 Hz) [17–19]. These abnormal patterns of brain activity are thought to disrupt normal network dynamics and synaptic plasticity, processes that are essential for cognitive functions such as memory consolidation and spatial navigation [7, 20, 21]. Notably, patients with epilepsy are at significantly higher risk of developing AD [22], but the underlying mechanisms remain poorly understood. Further studies are needed to investigate the relationship between changes in neural activities due to epileptiform events and cognitive decline in preclinical models of AD.

To address this knowledge gap with respect to the HFOs, IEDs, and cognitive decline, we used a preclinical AD model to explore the neural correlates of pathological brain signals and cognitive impairment in AD. We chose a rat model because of its much larger brains, cerebrospinal fluid and blood volumes, and higher intelligence. Additionally, rat models facilitate complex behavioral testing, regional imaging, electrophysiological recording, and fluid biomarker analyses that enhance understanding of their relationships to behavior deficits [23]. To test our hypotheses, we used a transgenic rat model that expresses APP and PS1 mutations but also has cerebral small vessel disease (chronic hypertension-driven cerebrovascular small vessel disease, a prevalent co-morbidity, that accelerates tauopathy in the model [24, 25]).

In our transgenic rat model featuring both SVD and Alzheimer’s pathology [24], we focused on characterizing the co-occurrence of IEDs and HFOs, evaluating their progressive development over time, and assessing their association with cognitive impairment. Furthermore, we aimed to explore the potential mechanisms underlying these pathological signals by analyzing the coupling between IEDs and HFOs and their contribution to cognitive deficits. By integrating electrophysiological recordings, immunohistochemistry, and behavioral assessments, the present study provides critical insights into the neural dynamics underlying memory decline in AD and lays the groundwork for identifying novel biomarkers and therapeutic targets.

## Method

### Animals

Longitudinal studies were conducted on female SHRFAD Alzheimer’s disease (FAD+) rats starting at 8 months of age (*n* = 24). Fourteen (*n* = 14) female littermates (FAD-) were also used. All experimental animals were female to reduce variability associated with sex-specific differences in hypertension observed in this background strain. Female rats were chosen due to their lower hypertension severity and better suitability for longitudinal studies. The SHRSPFAD Alzheimer’s disease rat model which was developed by breeding the APPswe/PS1ΔE9 transgenes into the stroke-prone spontaneously hypertensive rat (SHRSP) background [24], was maintain at the UCLA Division of Laboratory Animal Medicine (DLAM) and Veterans Affairs Greater Los Angeles Health Care System vivarium. All the experimental procedures were approved by the University of California, Los Angeles Institutional Animal Care and Use Committee (Protocol ID UCLA ARC-2019-073) and GLAVA IACUC (GOV.IRBNET #1750389) and carried out in compliance with the National Institutes of Health guide for the care and use of Laboratory animals. Rats were maintained at 12/12 hours light-dark cycle with access to environmental enrichment, standard food and water ad libitum. Transgene- negative littermates were used as controls.

**Fig. 1:**
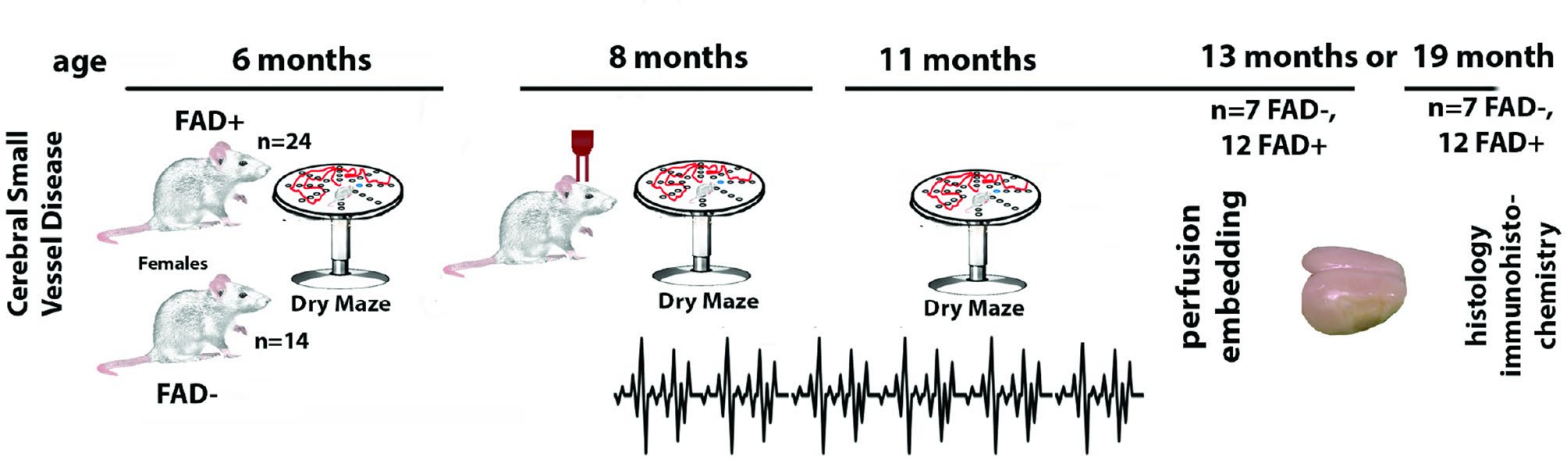
Experimental design and procedures. The experimental procedure. Day 0 indicates animal birth. At six months of age, all animals underwent the first behavioral experiment. At eight months of age, FAD-positive rats (*n* = 24) and transgene-negative littermates (*n* = 12) were bred onto an SHRSP (spontaneously hypertensive stroke-prone) background, modeling cerebral small vessel disease (CSVD). The second behavioral experiment was performed, and 16 depth electrodes were implanted for continuous neural signal recording. At eleven months of age, the third behavioral experiment was performed, with ongoing neural recordings. In both the 13- and 19-month-old groups, rats were transcardially perfused with physiological buffer; brains were immersion-fixed in formalin, paraffin-embedded, and sectioned for immunohistochemistry

The experimental design is illustrated in Fig. 1. In total, there are two cohorts of rats: cohort 1: Twenty-four (*n* = 24) rats and cohort 2: fourteen (*n* = 14) rats. At 8-months of age, cohort 1 rats were developed by breeding the APPswe/PS1ΔE9 transgenes into the stroke-prone spontaneously hypertensive (classified as FAD + group). The presence of human APPswe and PS1ΔE9 transgenes was confirmed by real-time PCR, as described by Cohen et al. [26]. Genotyping of rat ear biopsy samples was performed by Transnetyx Automated PCR Genotyping Services (www.transnetyx.com; Cordova, TN) using gene-specific probes. Forward, reverse and reporter primer sequences for these transgenes specific to this rat model are available upon request from Transnetyx. Cohort 2 rats remained unchanged (FAD- group). At this time point, both cohorts of rats (*n* = 14 from FAD + group, *n* = 14 from FAD- group) were implanted with 16 channel depth microelectrodes for brain signal recording. EEG recordings were continuously performed from month 8 to month 11 (total 8–10 weeks) of animal age. Behavioral experiments were performed at 6, 8, and 11 months of age.

At the end of the experiments, all rats in this study were euthanized either at 13 or 19 months of age to assess the association of electrophysiological changes with Alzheimer progression. Rats were first deeply anesthetized with isoflurane inhalation anesthesia, then injected with a lethal dose of 100 mg/kg body weight. After cessation of breathing, all rats were transcardially perfused with physiological buffer and the brain removed. Rats were then immersion-fixed with 10% formalin, followed by paraffin embedding and sectioning (8 μm) as previously described [24, 25].

### Experimental protocols

#### Behavioral experiment

A series of behavioral experiments were conducted across three age periods: 6 months of age, 8 months of age and 11 months of age. In all periods, a spatial memory test was administered to assess the animals’ learning and recall functions [27, 28]. A water-cheeseboard maze (WCBM) was employed on rats subjected to water deprivation. The weight loss of rats was no more than 5% after this. The experiment comprised two phases: on the first day, learning function was measured, while on the second day, recall function was measured. Each day, a total of ten trials were conducted, with each trial involving the placement of an animal in the starting box, door opening, and the allowance of the rat to search for a well containing water (water wells: unwater wells = 3:113). Upon locating all the water wells, the rat was returned to its starting box. All data were recorded and analyzed using AnyMaze software. The rats’ cognitive performance was assessed based on their ability to recall the spatial locations of three water wells, measured by the total time each subject took to locate them.

#### Electrode implantation electrophysiological data acquisition

All animals underwent electrode implantation at both eight months and eleven months of age. The following is a brief overview of the experimental procedures. The rats were first anesthetized using isoflurane (4–5% for induction and 1.5-2% for maintenance), and then fixed in a stereotactic surgery frame. The animal’s body temperature was maintained at 37 °C using a thermostatically controlled heating pad and their skin was parted, and the skull was exposed. Next, recording tungsten microelectrodes (50 μm outer diameter, with 1 mm space between tips) were implanted bilaterally in: (1) prefrontal cortex; AP = 3.7, ML = ± 0.5, DV = 3.0 (2) striatum; AP = 1.6, ML = ± 1.8, DV = 6.0 (3) anterior thalamus; AP = -1.40, ML = ± 1.0, DV = -6.5 and (4) hippocampus AP = -5.2, ML = ± 5.2, DV = 7.4. Ground and reference electrodes (stainless steel micro screws) were positioned in the cerebellum, 2.0 mm posterior to lambda. After implantation, the electrodes were secured with dental acrylic cement and the rats were placed in the heated cage for recovery. One week after surgery, the rats were connected to an EEG data acquisition system (Intan RHD 2000 digital system, Los Angeles, CA). Wide-band brain electrical activity (0.1 Hz to 3.0 kHz; sampling frequency = 3000 Hz) was recorded. EEG data were acquired continuously for 24 h per day, three days per week for 8–10 weeks.

### Immunohistochemistry

Ten µM Coronal paraffin sections were cleared, incubated on warmer at 60 degrees for 1 h, cleared with Citrosolvent, then rehydrated. Next, slides were warmed to room temperature for 1 h and steamed for 20 min using an antigen unmasking solution. Sections were quenched in 0.3% hydrogen peroxide (or 0.6% with 6E10) with methanol for 30 min, washed 3 times with tween buffer solution, note for 6E10 sections an additional step was needed. Sections were treated with 50% formic acid for 20 min. Then, all sections were then treated with 0.3% Triton X-100 in 0.1 M tris-buffered saline (TBS, pH 7.4) for 10 min at room temperature to enhance permeability. After pretreatment, sections were blocked with 3% bovine serum albumin (BSA) and 1.5% normal serum in TBS for 1 h at 37 °C to reduce non-specific binding. Primary antibodies used were as follows: mouse monoclonal anti Abeta 4–10 (6E10, 1:500, BioLegend, catalog # 803001, Covance #SIG-39320 ); rabbit polyclonal Phospho Tau-Thr217 (1:800), ThermoFisher catalog #44–744; rabbit polyclonal anti-neuropeptide Y (1:500), Cell Signal, catalog #11976; rabbit polyclonal 14-3-3-Gamma YWHAG (1:100), Cell Signal, Catalog #D15B7; mAb #5522; rabbit polyclonal iba1, (1:200) Wako Chemicals catalog #016-20001; mouse monoclonal GFAP (1:5000) for astrocytes, Sigma-Aldrich, catalog #G3893). Slides were also stained for H&E, Luxol Blue/Cresyl Violet and Congo Red/Hematoxylin to visualize general pathology and confirm genotype (not shown), using standard procedures. Following this, sections were incubated overnight at 4 °C with primary antibodies. The next day, sections were incubated with secondary antibodies (Goat anti-mouse HRP) diluted in normal serum and 3% BSA for 1 h at 37 °C, followed by avidin-biotin complex (ABC) reagent (Vector Labs) for 1 h 20 min at 37 °C. Immunoreactivity was visualized using metal-enhanced peroxidase diaminobenzidine (DAB; Pierce, Rockford, IL, United States) to detect pathology. Slides were then rinsed, dehydrated, and cover-slipped for imaging and analysis.

### Data analysis

#### Automatic detection of HFOs

The LFP data were firstly selected from the NREM and REM sleep period. Then, a two-step algorithm was implemented for the detection of HFOs, adapting from our previous publication [29]. The algorithm is outlined below: (1) An 80–520 Hz bandpass filter was applied, and the envelope of the filtered signal was detected. Epochs with significant higher amplitude than the baseline were kept for further analysis. (2) A time-frequency analysis was conducted to remove sharp transients. (3) A morphological analysis was introduced to discard the epochs with either low power level or with non-bulb shape on the time-frequency map. (4) Lastly, the HFOs was further cleaned from removing the events that were considered as the background signals.

#### Automatic detection of IEDs

The identification of IEDs was accomplished through the implementation of a four-step algorithm [30]. First, a band-pass filter within the 60–80 Hz range was employed to process the LFP signal, which necessitated resampling at a rate of 1250 Hz and conversion to a normalized squared signal (NSS). Second, the standard deviation (SD) of the baseline periods was calculated. The identification of IEDs was based on the presence of signals exceeding five times the SD above the mean NSS, and the elimination of IEDs events where the peak was less than 20 SD of the mean NSS baseline SD. The signal was subsequently restricted to the range of 30–250 milliseconds. Shannon Entropy correction was then applied to all events to eliminate shape transitions. Finally, all events were subjected to visual inspection and manual curation.

#### Coupling between IED and HFOs

To quantify the coupling strength between hippocampus IEDs and HFOs, cross-correlogram algorithm was first performed between these two electrophysiological signals and the peri-event time histogram (PETH) of synchronization was measured [31, 32]. For the PETH analysis, the input data consisted of timestamped occurrences of both IEDs and HFOs detected within the hippocampus, extracted from artifact-free segments of EEG recordings. Specifically, the timestamps of IEDs were used as anchor events (time = 0), and HFO occurrences within a ± 500 ms peri-event window were collected across all recordings to build a probability density histogram of HFO occurrence relative to IEDs. This approach enabled the identification of preferred temporal alignment of HFOs with IEDs, irrespective of the underlying event rate.

Then the Shannon Entropy correction was applied to measure the strength of the coupling through the PETH [33]. Specifically, for histogram with N bins, the $\:{\text{p}}_{\text{i}}$ was the probability of an event belonging to the $\:\text{i}$th bin, S defined as: $\:\text{S}=-{\sum\:}_{\text{i}=1}^{\text{N}}{\text{p}}_{\text{i}}\text{L}\text{n}\left({\text{p}}_{\text{i}}\right)$. Larger S means more uniform distribution, so we found the maximum S by $\:{\text{S}}_{\text{m}\text{a}\text{x}}=-{\sum\:}_{\text{i}=1}^{\text{N}}\frac{1}{\text{N}}\text{L}\text{n}\left(\frac{1}{\text{N}}\right)=\text{L}\text{n}\left(\text{N}\right)$. To examine the authenticity of measured strength, we applied bootstrapping [34, 35]. The strength $\:{\text{h}}_{\text{i}\text{j}}$ between two events was calculated by subtracting the Shannon entropy calculated for the peri-event time histogram: $\:{\text{h}}_{\text{i}\text{j}}=\frac{{\text{S}}_{\text{m}\text{a}\text{x}}-\text{S}}{{\text{S}}_{\text{m}\text{a}\text{x}}}$.

#### Statistics

##### Analysis of HFO and IED occurrence rate

All statistical analyses were performed using GraphPad Prism version 10.4.1. HFO occurrence rates (events/sec) were calculated for each brain region (striatum, medial prefrontal cortex, hippocampus, and thalamus) at two time points (8 and 11 months of age), corresponding to the behavioral testing periods. A similar approach was used to calculate IED rates across the same regions and time points. Group comparisons (FAD- vs. FAD+) and within-group comparisons across time points were analyzed using two-way repeated-measures ANOVA, with brain region and time point as within-subject factors and genotype as the between-subject factor. Post hoc pairwise comparisons were performed with Sidak correction to control for Type I error across multiple comparisons. Statistical significance was set at *p* < 0.05.

##### Analysis of the behavior data and its association with electrophysiological signals

Group differences in behavioral performance between FAD − and FAD + rats at 8 and 11 months were analyzed using two-way repeated-measures ANOVA, with age (time) as the within-subject factor and genotype as the between-subject factor. Post hoc pairwise comparisons used the Sidak correction for multiple testing. To examine the relationship between electrophysiological biomarkers (HFO rate, IED rate, and IED–HFO coupling) and cognitive performance, linear regression analyses were performed. Behavioral outcomes (task completion times) were treated as dependent variables, with hippocampal electrophysiological measures as independent variables. Pearson’s correlation coefficients (r) were calculated to assess the strength and direction of the linear relationships.

##### Statistical analysis of immunohistochemistry

Histology data were assessed using customized ImageJ plugins. For each antigen, microscopic images of DAB (purple) labeled antigens in brain regions were acquired with a Leica Microscope Camera, ensuring the same lighting and condenser settings. Once the antigen-specific image parameters for a specific antigen were set, they were incorporated into the plugin so that all the images for that antigen were processed identically. While the plugin first performs background subtraction to minimize lighting or staining artefacts, the experimenter sets the optical density thresholding parameters and particle size limitations before automating the analysis (for example to eliminate analysis of speckle artefacts). Antigen specific differences in parameters are as follows. For plaques, particle size exclusion of large and small plaques was incorporated into the plugin, while for GFAP staining patterns, where the process staining is light, and cell bodies are stained dark, the plugin analyzes them separately. For most other antigens that show subtle changes in darkness of staining, a single particle size and density thresholding range is needed to detect this change and incorporated into the plugin. Images were acquired from Bregma − 3.5 mm, a region with high pathology in multiple regions and the average of two images from the same animal was assessed. Statistical analysis was performed using IBM SPSS version 30.0, and graphs prepared with GraphPad Prism Version 10.4.1 for Mac Osx.

For regional and age dependent plaque deposition, a mixed model two-way ANOVA (Region × Transgene), adjusting for regions within each animal as a repeated measure, was performed to compare plaque sized, relative optical density and burden across different brain regions (entorhinal cortex, hippocampus, frontal cortex, and thalamus). Two-tailed unpaired t-tests were used to assess the transgenic effect on one parameter in one region, while two-way ANOVA (Age × Transgene) was used when assessing age and transgene effects in a specific brain region. Fisher’s Least Significant Difference (LSD) test was used for post hoc planned comparisons in ANOVA. Quantitative data were presented as mean ± SEM. Statistical significance was defined as *p* < 0.05.

## Results

### Neuropathological correlates with electrophysiological deficits

**Fig. 2:**
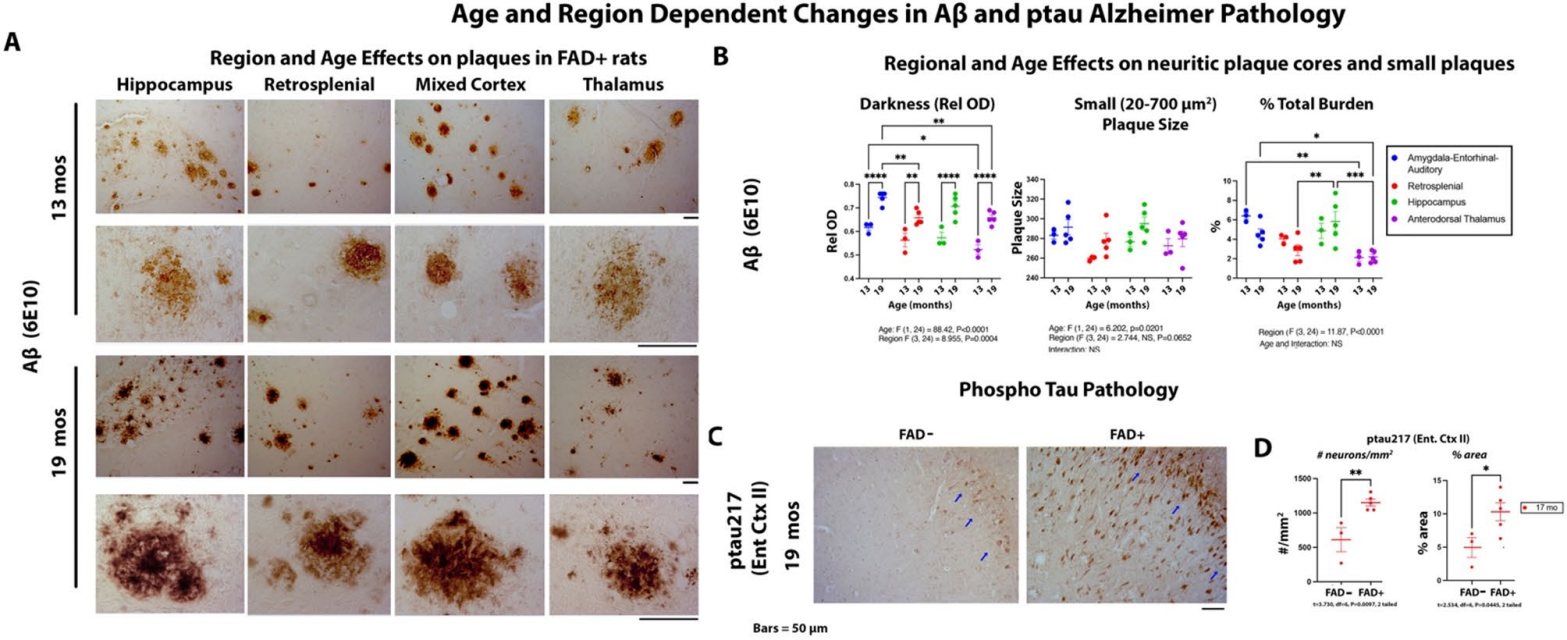
Age and region dependent changes in Abeta-ir plaques and ptau217 Alzheimer Pathology. **A & B.** Representative anti- Aβ (6E10)-stained brain sections showing age- and region-dependent changes in amyloid-β (Aβ) plaques in the (i) mixed cortex (amygdala, entorhinal cortex and auditory cortex) (ii), hippocampus (iii) retrosplenial cortex and (iv) anterodorsally thalamus at -3.6 mm Bregma. At 13 months of age, amyloid plaques lacking compact cores “cotton wool plaques” were predominant, while at 19 months neuritic plaques with dark cores prevailed. **B**. Age-related patterns in plaque deposition were assessed using mixed two-way ANOVA adjusting for within animal regional changes used as repeated measures. The compaction of the plaques was also illustrated by changes in the relative optical density (Age: F (1, 24) = 88.42, *P* < 0.0001, Region F (3, 24) = 8.95, *P* = 0.0004). (**B**), Despite no significant change in the size of large plaques (> 700 µm^2^, not shown), there was a strong age dependent increase in small plaques (Age: F (1,24) = 6.20, *p* < 0.001, which was unexpectedly, region independent, but most notable in the hippocampus. For total percent burden, strong regional differences were seen (Region: F (3,24) = 11.87, *p* < 0.0001); planned LSD post hoc comparisons showed the main regional change at 13 months was due to high burden in the mixed cortex, while by 19 months there was more plaque accumulation in the hippocampus. As the cotton wool plaques developed dense cores with age, the total percent amyloid burden was surprisingly reduced in the mixed and retrosplenial cortex but did not reach significance. (**C)** and (**D)** show micrographs and quantitative evaluation of ptau pathology. An Abeta sensitive phospho tau (ptau) site 217 was stained. At 13 months of age there were no transgene effects on ptau 217 in any regions (not shown). However, by 19 months of age, the Entorhinal Cortex layer 2 (E.Ctx II, blue arrows), the first site of tau pathology in human AD, showed increase ptau burden and number of neurons stained per area (**D**). Data show that both the number per region (t = 3.73, df = 6, *p* = 0.0097) and tau burden (t = 2.53, df = 6, *p* = 0.044) were increased with age in FAD + rats. Magnification bars represent 50 μm. Data are presented as mean ± SEM, with statistical significance indicated as ∗*p* < 0.05, ∗∗*p* < 0.01

**Fig. 3:**
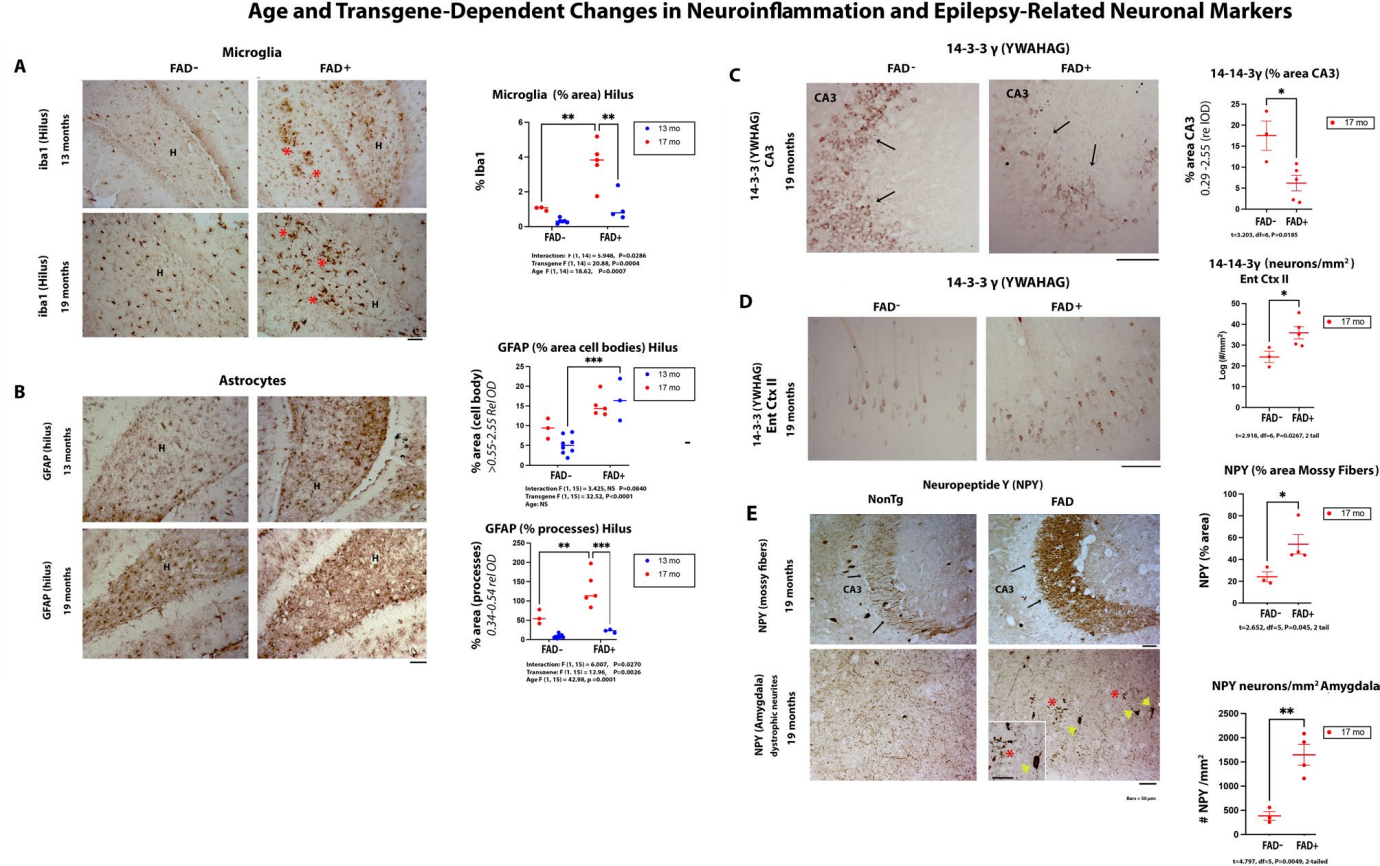
Age and transgene dependent increases in neuroinflammation and epilepsy related biomarkers. **A.** Microglia were stained with iba-1. The micrograph panel shows the microglial staining pattern in the hilus (“H”). Like AD brain, in the FAD + rat, microglia cluster around neuritic plaques (**red asterisks**), but also increase in the neuropil independent of plaques. Analysis of percent staining shows that there is disproportionate increase in microglia with age in FAD + rats as illustrated by a strong transgene F (1, 14) = 20.88, *p* = 0.0004 and age F (1, 14) = 18.62, *p* = 0.0007 effects and interaction between transgene and age F (1, 14) = 5.948, *p* = 0.0286. **B**. Astrocytes staining (GFAP) in the hilus “H”. The cell bodies showed a strong transgene effect (F (1, 15) = 15.39, *p* = 0.0014 without an age effect. However, indicative of astrocyte hypertrophy, the GFAP processes not only displayed a strong transgene effect (F (1, 15) = 6.007, *p* = 0.027), but also showed a large age effect (F (1, 15) = 42.98, *P* < 0.0001) and a transgene-age interaction (F (1, 15) = 12.96, *p* = 0.002). (**C**) and (**D)** show evaluation of two epilepsy-related biomarkers, which can have protective compensatory as well as detrimental roles. **C**. Transgene dependent reduction in the percent area stained for 14-3-3 γ was significant in the hippocampal CA3 (**black arrows**) neurons by 19 months of age (t = 3.203, df = 6, *p* = 0.0185). **(D)** Neuropeptide Y (NPY) distribution was also examined. Like observations in mouse models of FAD with seizures, Neuropeptide Y was found to accumulate in the mossy fibers (**black arrows**) in 19-month rats with FAD+ (t = 2.652, df = 5, *p* = 0.0453). A very different pattern of NPY staining emerged in the amygdala where the transgene dependent changes manifested in NPY staining of plaque (**yellow arrowheads**) associated dystrophic neurites (**red asterisks)** seen at higher magnification in the (t = 4.797, df = 5. *p* = 0.0049) with statistical significance indicated as ∗*p* < 0.05, ∗∗*p* < 0.01

We examined the brains of rats in this study to track the trajectory of progression in various biomarkers for AD and epilepsy. We evaluated AD pathology as shown in Fig. 2, which describes the age- and region-specific changes in Aβ-immunoreactive (Aβ-ir) plaques and pTau217 pathology. Figure 2A presents micrographs showing age-related Aβ plaque deposition across four brain regions: (i) mixed cortex, (ii) hippocampus, (iii) retrosplenial cortex, and (iv) anterodorsally thalamus. By 13 months, amyloidogenic plaques lacking a dense core—commonly referred to as “cotton wool” plaques—predominate. By 19 months, these plaques appear to evolve into neuritic plaques with dense cores. Figure 2B quantifies these changes, demonstrating a significant age-related increase in plaque compaction (*p* < 0.0001). Interestingly, while the size of large plaques remained unchanged (data not shown), the number of small plaques significantly increased with age (*p* < 0.001), particularly in the hippocampus. Total plaque burden analysis highlighted significant age and region interactions, with the mixed cortex showing disproportionately high plaque accumulation at 13 months, whereas the hippocampus exhibited preferential plaque deposition by 19 months. Figures 2C **and D** depict tau pathology using antibodies targeting the Aβ-responsive phosphoepitope pTau217. At 13 months, no transgene-related tau pathology was observed (data not shown). However, by 19 months, pTau217 accumulation was evident in the entorhinal cortex layer II of FAD + rats (blue arrows), resembling early tau pathology seen in human AD. Figure 2D quantifies this progression, revealing a significant increase in both pTau217 burden and the number of pTau217-positive neurons in this FAD-sensitive neuronal layer (*p* < 0.001). **We also examined other neuropathological markers common to AD and epilepsy.** Figure 3 highlights the interplay between neuroinflammation, epilepsy-related biomarkers, and transgene expression over time. Figure 3A shows progression in microglial activation, evidenced by Iba-1 staining. Microglia clustered around neuritic plaques (red asterisks) in the hilus (H) of FAD + rats, a hallmark of Alzheimer’s pathology. Microglial density increased significantly across the neuropil, with strong age and transgene effects, as well as age-transgene interactions, suggesting accelerated neuroinflammation by 19 months. Figure 3B examines astrocyte reactivity using GFAP staining. While both age and transgene effects were significant, the staining pattern differed from microglial activation. In the hilus, gliosis appeared to have plateaued, characterized by hypertrophic astrocytic processes. As a result, the evaluation of astrocyte cell bodies primarily revealed a transgene effect, whereas the analysis of astrocytic processes demonstrated significant age, transgene, and interaction effects, with astrocyte hypertrophy escalating several-fold in aging FAD + rats compared to transgene-negative littermates. Figure 3C investigates changes in 14-3-3γ, a neuronal protein implicated in epilepsy. Staining was predominantly cytosolic in CA3 pyramidal neurons of both FAD + and transgene-negative rats. While no differences were observed at 13 months (not shown), by 19 months, 14-3-3γ levels were significantly reduced in CA3 neurons of FAD + rats (black arrows, *p* = 0.0185), suggesting network instability. Figure 3D shows that compared to FAD- rats 14-3-3 in the entorhinal cortex layer II neurons, was also not impacted at 13 months of age (not shown), but at 19 months was significantly increased, in the opposite direction compared to in the hippocampus [36]. Figure 3E demonstrates that by 19 months, FAD + rats exhibited Neuropeptide Y (NPY) accumulation in CA3 mossy fibers—a phenomenon observed in human epilepsy and rodent models. Additionally, in another region, the amygdala, the NPY staining was associated with dystrophic neurites surrounding plaques. Collectively, Figs. 2 and 3 underscore progressive AD pathogenesis, neuroinflammation and epilepsy-related changes in FAD + rats, shedding light on their role in disease progression.

### Alterations in HFOs in FAD + rats

**Fig. 4:**
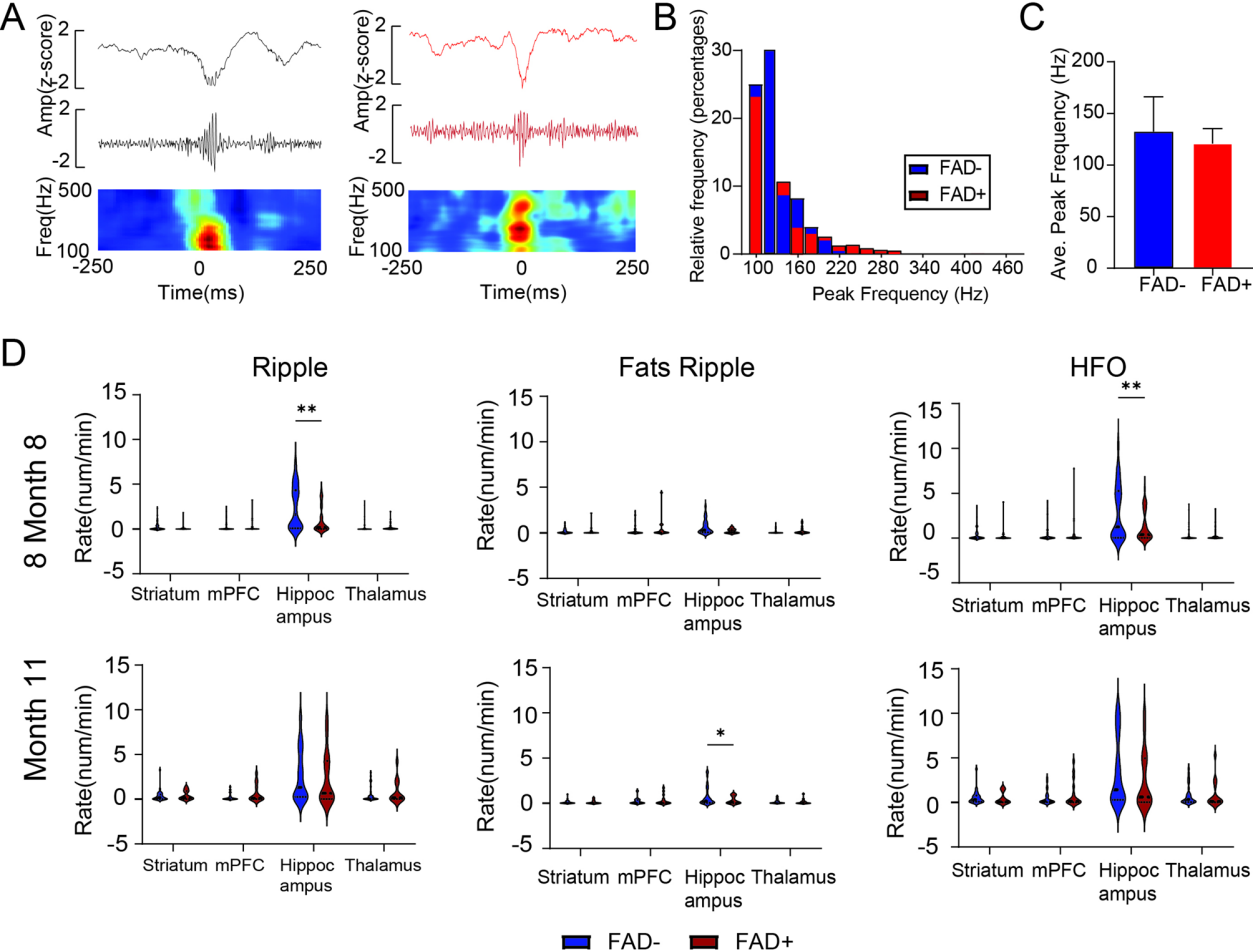
FAD + transgene and region-specific electrophysiological measurements of HFOs at 8 and 11 months of age. **(A)** Illustrations of HFO events (black: ripple; red: fast ripple) with unfiltered LFPs, filtered traces (100–500 Hz) and time-frequency plots. **(B)** Peak frequency distribution of HFO events in FAD− (blue) and FAD+ (red) rats. **(C)** Average peak frequency of HFO events in FAD− (*n* = 13, mean = 132 Hz) and FAD+ (*n* = 12, mean = 121 Hz) groups. **(D)** Region-specific occurrence rates of HFOs, ripples, and fast ripples (events/min) in FAD+ (red) and FAD− (blue) rats at 8 months (top) and 11 months (bottom) of age. Two-way ANOVA revealed significant group differences. 8 Months: Ripple rates were significantly reduced in the hippocampus of FAD + rats (t = 6.7, df = 384, *p* < 0.001). No significant group differences were observed for fast ripples. HFO rates were significantly lower in the hippocampus of FAD + rats (t = 5.67, df = 384, *p* < 0.001). 11 Months: No significant group differences were detected except for fast ripples in the hippocampus (t = 2.4, df = 178, *p* = 0.017). Statistical significance is indicated as *p < 0.05, **p < 0.01

We analyzed a total of 333 h of electrographic data from 14 FAD + rats and 14 FAD- rats (*n* = 28) (Fig. 1 and Supplementary Figure S4). Behavioral and electrographic seizures were recorded in three out of 14 FAD + rats, while no seizures were observed in the FAD − group. HFOs were analyzed by separating ripples and fast ripples (Fig. 4A). We first examined LFP-derived HFO properties across four brain regions: striatum, prefrontal cortex, hippocampus, and thalamus. To compare HFO rates between groups, we detected HFO peaks in FAD − and FAD + rats (Fig. 4B). There was no difference in average peak frequency of HFOs between the two groups (Fig. 4C).

Group-wise comparisons of HFO, ripple, and fast ripple rates were conducted at both 8 and 11 months of age. At 11 months, only the fast ripple rate in the hippocampus was significantly higher in FAD + rats (*p* = 0.017) (Fig. 4D). In contrast, at 8 months, significant differences were observed for HFO and ripple rates in the hippocampus (HFO: *p* < 0.001; ripple: *p* < 0.001) as well as in the prefrontal cortex (HFO: *p* = 0.038; ripple: *p* = 0.026) (Fig. 4D). No significant differences in fast ripple rates were detected at 8 months, and no differences were observed in the striatum, mPFC, or thalamus for any event type at either age (Fig. 4D). At 11 months, there were no overall significant differences in HFO or ripple rates across the four regions. Overall, these findings suggest that HFO and ripple rates were reduced in the FAD + group, but this difference was limited to 8-month-old animals.

All analyses above were performed using NREM sleep-stage data, given its relevance to memory consolidation processes. However, because epileptiform activity and HFOs can vary across sleep-wake states, we also conducted additional analyses of REM sleep-stage data to evaluate whether these findings generalized beyond NREM sleep. A total of 13.4 h of REM recordings were analyzed from 17 animals (FAD−: 7.6 h from 8 animals; FAD+: 5.8 h from 9 animals), yielding 583 IEDs and 11,208 HFOs in total.

In REM sleep, there were no significant differences in HFO rates between FAD − and FAD + rats in any of the four brain regions at either 8 or 11 months of age. In contrast, consistent with the NREM findings, IED rates were significantly higher in FAD + rats across all brain regions at both ages (8 months: Striatum: t = 5.46, df = 88, *p* < 0.001; mPFC: t = 4.78, df = 88, *p* < 0.001; Hippocampus: t = 4.5, df = 88, *p* < 0.001; Thalamus: t = 5.76, df = 88, *p* < 0.001; 11 months: Striatum: t = 2.65, df = 73, *p* = 0.039; mPFC: t = 2.78, df = 73, *p* = 0.027; Hippocampus: t = 3.65, df = 73, *p* = 0.002; Thalamus: t = 3.41, df = 73, *p* = 0.004) (Supplementary Figure S3).

### Increased IED rates and enhanced IED–HFO coupling in FAD + rats

**Fig. 5:**
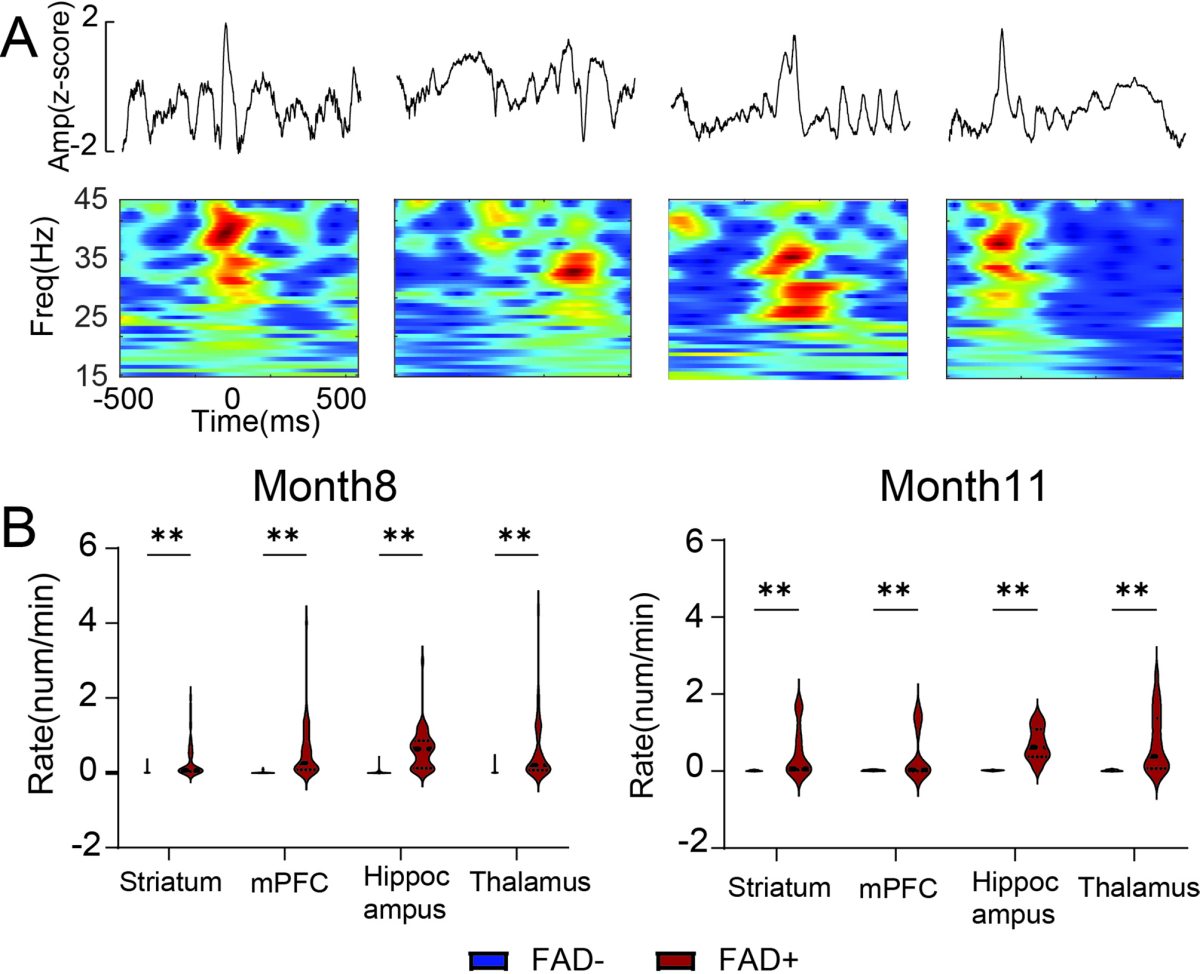
FAD + transgene and region-specific electrophysiological measurements of IEDs at 8 and 11 months of age. **(A)** Illustrations of the IEDs detected (showing unfiltered LFP (top) and time-frequency plot (bottom)). **(B)** Left: Region-specific occurrence rates of IEDs (events/min) in FAD+ (red) and FAD− (blue) rats at 8 months of age. Significant increases were observed in FAD + rats: Striatum (t = 4.1, df = 508, *p* < 0.001), mPFC (t = 7.22, df = 508, *p* < 0.001), Hippocampus (t = 8.15, df = 508, *p* < 0.001), and Thalamus (t = 7.03, df = 508, *p* < 0.001). Right: Region-specific occurrence rates of IEDs at 11 months of age. Significant increases in FAD + rats were found in all regions: Striatum (t = 4.58, df = 232, *p* < 0.001), mPFC (t = 3.89, df = 232, *p* < 0.001), Hippocampus (t = 7.32, df = 232, *p* < 0.001), and Thalamus (t = 7.84, df = 232, *p* < 0.001). Statistical significance is indicated as *p < 0.05, **p < 0.01

We next performed IED analysis (Fig. 5A), as these events have been identified as a significant electrophysiological feature in mouse AD models [3]. The results revealed significant differences between the FAD + and FAD − groups across all four brain regions, with the FAD + group exhibiting a considerably higher IED rate (*p* < 0.001) during both the 8-month and 11-month recordings (Fig. 5B). This finding indicates that FAD + rats exhibit a substantially higher incidence of IEDs, suggesting that increased IED activity in the FAD + group may contribute to emerging memory impairment, as validated by our behavioral tests at 11 months.

**Fig. 6:**
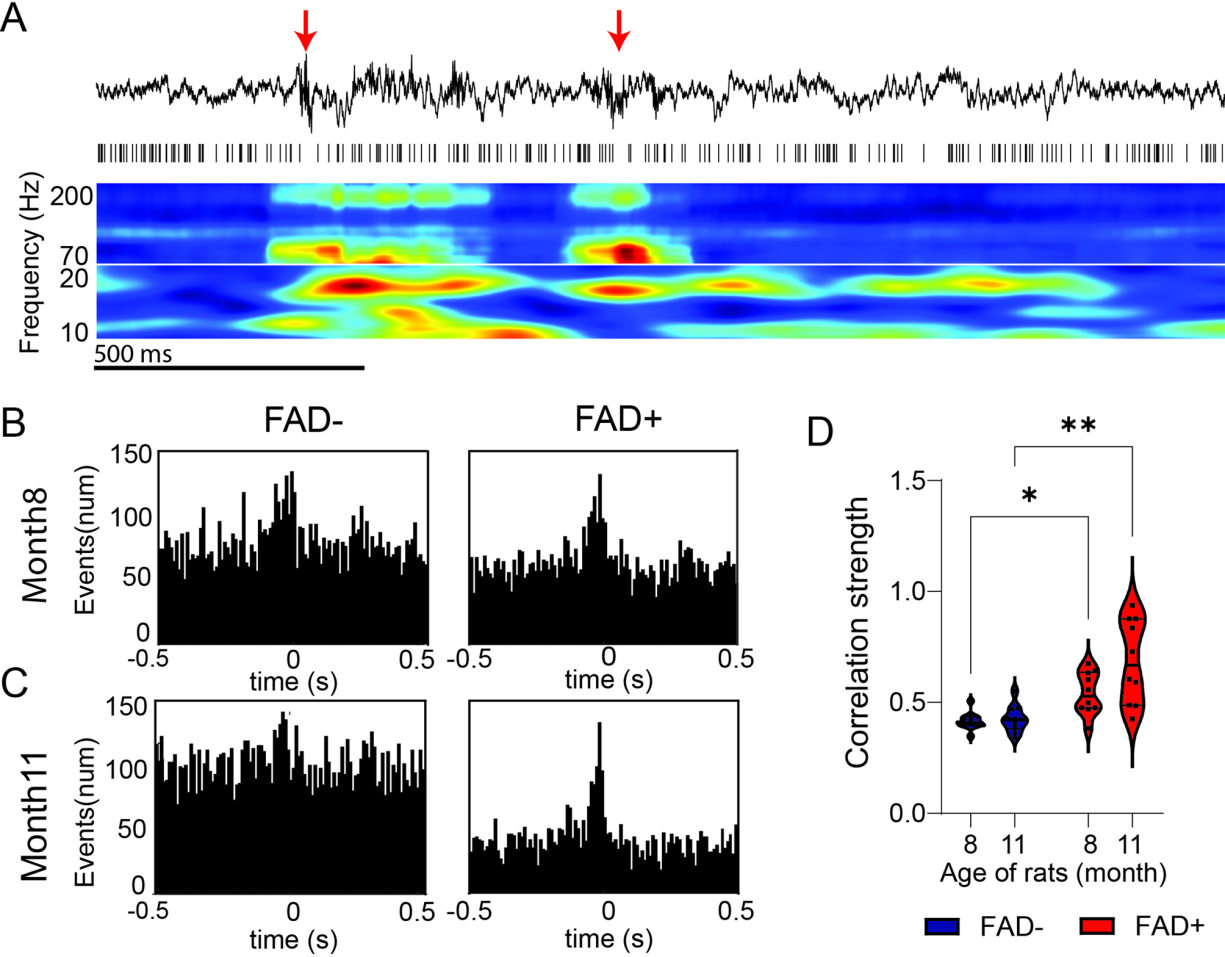
Age- and transgene-dependent changes in frequency, event counts, and coupling between HFOs and IEDs in the hippocampus. **A.** Representative unfiltered LFP segment (top), raster plot (middle), and time–frequency plot (bottom; 10–20 Hz and 20–200 Hz) from a selected channel. Red arrows show the locations where HFO-IED coupling occurs. **B–C.** Peri-event time histograms showing synchronization between hippocampal IEDs and HFOs in rats at 8 months (upper panels) and 11 months (lower panels) of age. The Y-axis indicates the number of HFO and IED events, and the X-axis indicates time relative to the IEDs. In panel B (8 months), event counts were: FAD− (left; HFO: *n* = 14,390; IEDs: *n* = 4,058) and FAD+ (right; HFO: *n* = 21,461; IEDs: *n* = 11,397). In panel C (11 months), event counts were: FAD− (left; HFO: *n* = 8,191; IEDs: *n* = 7,683) and FAD+ (right; HFO: *n* = 19,537; IEDs: *n* = 5,650). Statistical differences were assessed using two-way ANOVA (Transgene × Age) with rat ID as a factor, followed by planned comparisons using Fisher’s LSD. **D.** Coupling strengths between hippocampal IEDs and HFOs at 8 and 11 months in FAD− (*n* = 11) and FAD+ (*n* = 10) groups. The transgene effect was significant (F(1,38) = 3.75, *p* < 0.001). Post hoc analysis showed increased coupling in FAD + rats at 8 months (*p* = 0.025) and 11 months (*p* < 0.001) compared to FAD−. Statistical significance is indicated as *p < 0.05, **p < 0.01

Given the substantial disparities observed in IED and HFO rates in FAD + rats, we further examined the presence of any differences in IED–HFO synchronization, with a specific focus on the hippocampus, the region with the highest number of IEDs and HFOs (Figs. 4D and 5B). We consistently observed a characteristic pattern in the EEG data in which hippocampal IEDs appeared to trigger HFOs (Fig. 6A), motivating a more detailed exploration of their interaction. To analyze the coupling between hippocampal IEDs and HFOs, a Shannan Entropy approach was applied [33]. Quantitative analysis demonstrated clear synchronization between IEDs and HFOs in the FAD + group (as verified in the PETH, Fig. 6B and C) at both 8 and 11 months, suggesting a strong association between these two events. This association was less pronounced in the FAD − group. Statistical analysis revealed that the coupling strength between IEDs and HFOs in the FAD + group during both 8-month (*p* = 0.025) and 11-month (*p* < 0.001) recordings was significantly higher than in the FAD − group (Fig. 6D). Moreover, this trend became more pronounced as animals aged.

### Neural correlates of memory impairment and cognitive deficits in FAD + rats

**Fig. 7:**
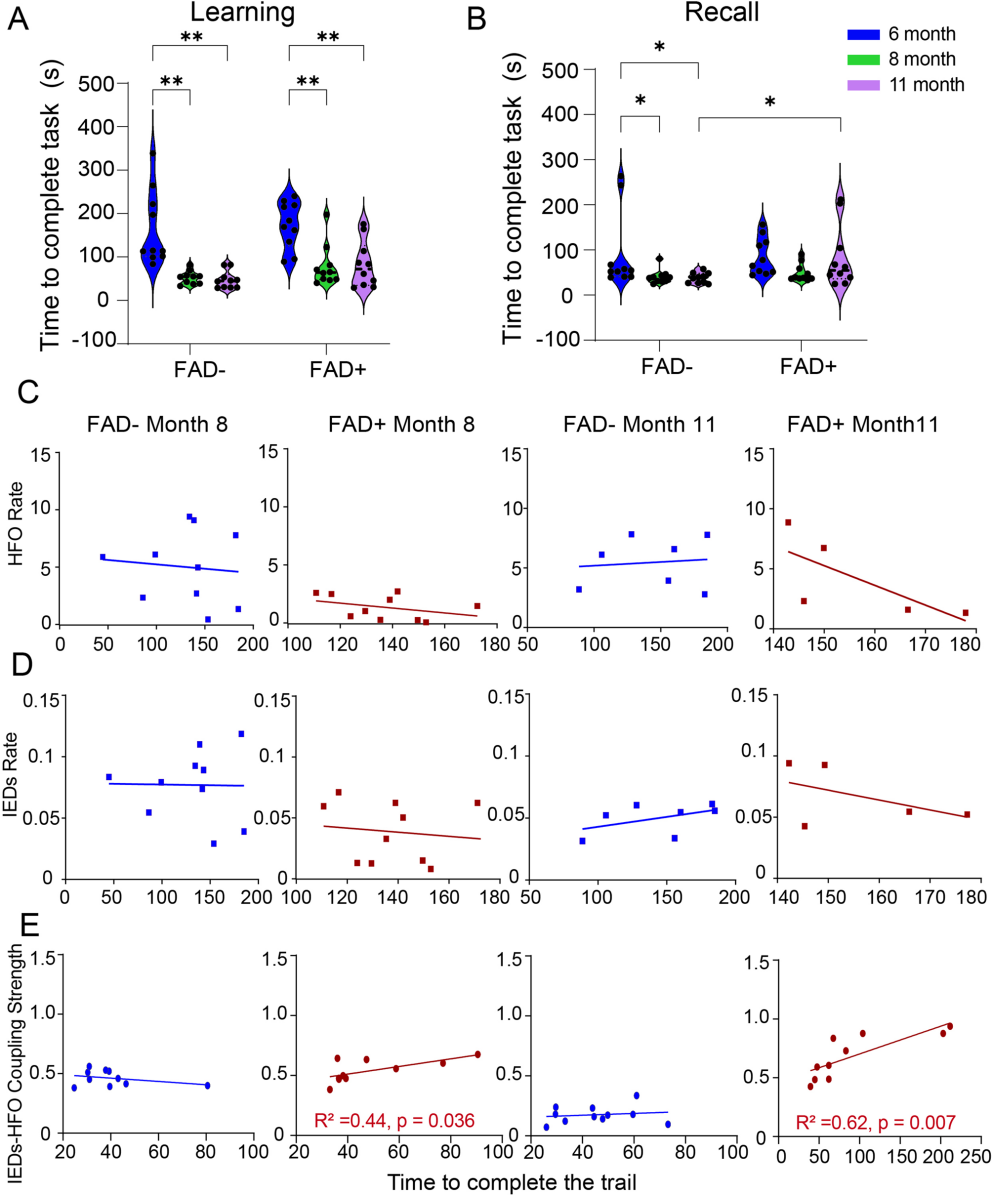
Relationship between hippocampal neuro-signal coupling and memory performance. **A & B**. Task completion times for learning (left) and recall (right) phases across ages. Colors indicate age groups: blue = 6 months, green = 8 months, purple = 11 months. Two-way mixed ANOVA was performed with time and within-animal changes as factors, followed by planned comparisons using Fisher’s LSD to assess genotype-specific cognitive differences. **(A)** Learning phase. Significant transgene effect (F(2,54) = 27, *p* < 0.001). Post hoc analysis showed differences in FAD − rats between 6 and 8 months (*p* < 0.001) and between 6 and 11 months (*p* = 0.001). Similar differences were observed in FAD + rats between 6 and 8 months (*p* < 0.001) and between 6 and 11 months (*p* = 0.001). **(B)** Recall phase. Significant transgene effect (F(2,54) = 4, *p* = 0.024). Post hoc analysis showed differences in FAD − rats between 6 and 8 months (*p* = 0.048) and between 6 and 11 months (*p* = 0.037). No significant differences were detected in FAD + rats. **C–E.** Regression analyses between memory performance scores (MPS) and electrophysiological measures. **(C)** HFO rate. At 8 months, no significant correlations in FAD− (R² = 0.83, *p* = 0.011) or FAD+ (R² = 0.14, *p* = 0.29) groups. At 11 months, no significant correlations in FAD− (R² = 0.012, *p* = 0.82) or FAD+ (R² = 0.52, *p* = 0.17) groups. **(D)** IED rate. At 8 months, no significant correlations in FAD− (R² = 0.00029, *p* = 0.96) or FAD+ (R² = 0.016, *p* = 0.73) groups. At 11 months, no significant correlations in FAD− (R² = 0.24, *p* = 0.27) or FAD+ (R² = 0.25, *p* = 0.39) groups. **(E)** IED–HFO coupling strength. Significant positive correlations were observed in the FAD + group at both 8 months (R² = 0.44, *p* = 0.036) and 11 months (R² = 0.62, *p* = 0.007). No significant correlations were observed in FAD − rats (8 months: R² = 0.11, *p* = 0.34; 11 months: R² = 0.024, *p* = 0.65). Statistical significance is indicated as *p < 0.05, **p < 0.01

The above observations of abnormal EEG patterns motivated us to further explore the effects of IED–HFO coupling on memory performance in animals with and without AD pathology. We conducted three sessions of behavioral tasks on rats at 6 months (consider as baseline), 8 months and 11 months of age for both learning and recall phases. The results showed that learning ability in both FAD − and FAD + rats improved with repeated testing, as evidenced by shorter times to complete the task in later sessions (Fig. 7A; FAD−: month 6 vs. month 8, *p* < 0.001; month 6 vs. month 11, *p* < 0.001. FAD+: month 6 vs. month 8, *p* < 0.001; month 6 vs. month 11, *p* < 0.001). There were no between-group differences in learning scores in any of the sessions.

In the recall phase, the FAD − group performed significantly worse at month 6 compared to later sessions, possibly reflecting improvement with repeated exposure (Fig. 7B; FAD−: month 6 vs. month 11, *p* = 0.048; month 8 vs. month 11, *p* = 0.037). In contrast, the FAD + group showed no improvement in recall performance with repeated testing. Additionally, the FAD + group performed significantly worse than the FAD − group at month 11 (*p* = 0.047) (Fig. 7B). These data indicate a decline in recall function as FAD + pathology progresses, a conclusion also supported by individual trial performance analyses (Supplementary Figures S1 & S2).

Based on these findings, a key question was whether the decline in performance during the recall phase and the absence of differences between earlier and later trails were influenced by the abnormal neural signals found in FAD + animals. To address this question, the neural correlates were investigated by regressing the electrophysiological measures (HFO rate, IED rate, and IED–HFO coupling strength) on memory performance in two test sessions (month 8 and 11). The results indicated no significant correlations between IED rate or HFO rate and behavioral performance in any group at either age (Fig. 7C and D). However, we found a positive relationship between IED–HFO coupling strength and recall performance scores in the FAD + group at both ages (Fig. 7E: month 8: *p* = 0.036, R² = 0.44; month 11: *p* = 0.007, R² = 0.62). In contrast, there was no significant correlation between IED–HFO coupling and recall performance in the FAD − group (Fig. 7E).

## Discussion

Consistent with AD mouse models, the presence of altered epileptiform physiological patterns, specifically HFOs and IEDs, are associated with cognitive impairment in our rat model of AD. This exacerbation of epileptiform patterns is particularly evident in tasks that assess memory, attention, and learning abilities [16, 37, 38]. Our data provide a more comprehensive and in-depth understanding of the association of electrophysiological changes with cognitive performance and progression of neuropathology. Although several previous studies have already provide in depth knowledge of associations of AD and epilepsy, including the role of hyperexcitability and network dysfunction in disease progression, a comprehensive understanding of the underlying mechanisms remains elusive [38–41].

Our findings advance the field by demonstrating a more detailed relationship between the coupling of hippocampal IEDs and HFOs and the occurrence of seizures. Although seizures in our study were observed only in 3 out of 14 rats (~ 21%), the actual number of seizures could be much higher because we did not record 24 h/day. Patients with AD often exhibit epileptiform activity that is subclinical and associated with earlier onset AD [16, 42], raising the question as to whether this aberrant activity is sufficient to precipitate cognitive decline. Previous studies have shown that APP mice can exhibit seizures; our findings in FAD + rats extend this work by suggesting that high-frequency oscillations and epileptiform activity—even in the absence of overt seizures—may contribute to cognitive impairment. An increased coupling of IEDs and HFOs is associated with the cognitive deficits in a transgenic AD rat model. Specifically, the results highlight significant distinctions between the FAD + and FAD- groups across a range of pathological electrophysiological patterns and cognitive performance assessments, thereby offering insights into the potential mechanisms through which aberrant hippocampal hyperexcitability, impaired synaptic inhibition due to reduce the interneuron activity, and disrupted IED–HFO coupling contribute to network instability and cognitive deficits. These findings provide critical insights into the underlying cognitive dysfunction in AD model rats, which develop more complete plaque, tangle, and neurodegenerative AD pathology than AD model mice expressing mutant APP and plaque pathology.

### Association of electrophysiological abnormalities with AD and seizure related neuropathology

The electrophysiological abnormalities observed with age corresponded to robust changes in plaque pathology both across age and brain regions. The cotton wool plaques (lacking dense cores) that predominated at 13 months age are characteristic of Congo amyloid angiopathy and presenilin mutations [43], but by 19 months of age, transformed into classic neuritic plaques with dense compacted cores. The increased regional differences at older age reflected disproportionate growth in the hippocampus and mixed cortex, a pattern typical of amyloid deposition in both human patients with AD and transgenic rodent models of AD [44–46]. This pattern has also been observed in AD pathology studies [47–50]. It remains unclear how age-related amyloid sequestration impact pathogenesis, whether positively or negatively. Notably the emergence of seizures in humans and animal models often occurs in the prodromal period [51, 52]. However longitudinal studies shown that seizures can occur throughout the course of AD [42].

Other neuropathological markers were also examined. There were age-dependent changes in microglial neuroinflammation, which increased exponentially in the hilus of FAD + rats at 19 months compared to their negative littermates. Microglia may influence HFOs [53], but a role for astrocytes is more commonly accepted. In the hilus, despite no detectable change in the number of astrocyte cell bodies between 13 and 19 months, their processes exhibited hypertrophy, leading to congestion of surrounding cells, with significant age-related effects and age-transgene interactions. The striking spatial expansion of these processes may contribute to seizure activity, as astrocytes play a critical role in seizure generation [54, 55]. In addition to inflammation other pathologies may contribute to seizure vulnerability. We examined 14-3-3 gamma (YWHAG), a protein emerging in GWAS and proteomic studies related to both AD and epilepsy [56, 57]. This previously unexplored link between AD and epilepsy pathogenesis is known to regulate mitochondrial energy metabolism, caspase activation, and cell death, as well as playing a role in modulating tau aggregation [58]. Data showed a loss of 14-3-3 gamma (YWHAG), in the CA3 neurons may increase vulnerability to excitotoxicity. Finally we also examined neuropeptide Y (NPY), a well-established marker of hippocampal hyperexcitability and epileptic activity in AD mouse models NPY has been reported to dramatically increase in mossy fibers in association with seizure activity [59, 60]. Our findings revealed a similar elevation by 19 months of age. In addition to the CA3 changes, we also observed NPY localization to dystrophic neurites surrounding plaques. This phenomenon was first reported in human AD plaques in 1985 [61], yet NPY is not widely recognized as a biomarker for dystrophic neurites in AD. Given its known role in mossy fibers, our findings suggest a stronger connection to epilepsy-related AD.

In addition to the entorhinal cortex playing being the initial site for tau pathogenesis in human during the prodromal phase, it is also important role in seizure generation as well as AD [62].

### Alterations in HFOs in FAD + Model

In the FAD + group, an increased rate in both HFO and ripple was observed in the striatum, prefrontal cortex, and hippocampus at 8 months of animal age. This finding aligns with previous studies that indicated HFO play a role in the early stage of AD pathology [4, 63–65]. In the early stage of AD pathology, there is more HFO and ripple in FAD- rats but not for fast ripples. the elevated HFO rate may be associated with the heightened neural excitability of dysfunction in the hippocampal-cortical circuits [66, 67]. One surprising finding is that there was no significant difference of both HFO and ripple rate between the tested groups at month 11. A potential explanation for these negative findings is that AD is a progressive disease [68] and HFO contains both ripples and fast ripples [69, 70]. More importantly, HFO generated through synchronized neuronal firing activity, particularly involving inhibitory interneurons and excitatory pyramidal cells [70, 71] are thought to play a crucial role in both physiological process, such as memory consolidation, and pathological conditions, particularly epilepsy and AD [4]. Their occurrence is highly dependent on the structural integrity of neuronal networks, with ripples primarily emerging from coordinated inhibitory-excitatory interactions and FRs often associated with hyperexcitable and pathological circuits [72–74]. Recent reports argue that with neurodegenerative conditions or epilepsy-related brain damage as the disease progresses, neuronal loss also occurs, and the reduction in excitatory-inhibitory balance alters the generation of HFOs, it eventually leads to decreased HFO occurrences, likely due to interneuron functional responses [17, 19]. As neuronal populations deteriorate, the altered network dynamics can result in the rebalancing of ripple and FR rates, causing previously observed group differences in oscillatory activity to disappear [75]. It remains to be shown whether there is a loss of neuronal populations or synaptic remodeling that not only reduces the number of HFOs, but also disrupts the mechanisms that differentiate physiological from pathological oscillations, leading to a convergence of ripple and FR frequencies across affected groups.

### IEDs as potential drivers of cognitive decline

Interestingly, we found that FAD + rats had significantly higher rates of IEDs in all brain regions, with the highest rate in the hippocampus. One possible explanation could be related to emergence of IEDs there as it has recently been shown in AD mouse lines that IEDs may start in hippocampal dentate gyrus using CSD analysis [76]. IEDs have been observed in a variety of clinical contexts, including in patients with epilepsy and AD, and are considered as a key biomarker of pathological brain activity in these conditions [3, 77]. In recent studies, IEDs were found to contribute significantly to cognitive impairments in both epilepsy and neurodegenerative disease, including AD [16, 30, 78, 79]. This pathological pattern is believed to disrupt the normal oscillatory activity and connectivity within critical brain regions, impairing cognitive functions such as memory, attention, and executive function [3, 30].

Consistent with a known role of IEDs in AD mice model [3, 80], our data go further, support the notion that epileptiform activity, including IEDs, plays an important role in AD and may exacerbate cognitive decline by disrupting normal neural processing and network stability [81]. The most significant findings were in the hippocampus. The hippocampus, a region pivotal for memory processing, may be particularly vulnerable to such disturbances [82, 83], as IEDs have been shown to interfere with the formation and retrieval of memories [30]. Our findings suggest that IEDs may not only serve as an indicator of neuronal dysfunction but may also act as a driver of cognitive decline in AD, consistent with the hypothesis that epileptic-like activity contributes to the cognitive impairment observed in patients with AD [81]. In contrast to HFO changes, both month 8 and month 11 showed similar significant increases in the rate of IEDs in the FAD + group. These data suggest that IEDs may represent an early continuous pathological feature in AD, potentially reflecting early or prodromal neuronal dysfunction and network disintegration that persists. Interestingly, the neuropathological biomarkers do show rapid and longitudinal deterioration, but the IEDs do not progress, which raises the question as to whether the epileptiform changes in early stage, set off an irreversible cascade of neurodegeneration [84–86]. Specifically, the relationship between IEDs and cognitive function has been well-documented in epilepsy [79, 87]. Based on our results, IEDs may have a similar deleterious impact on memory and behavior in FAD + rats due to the aberrant coupling within the hippocampus area [30].

### IED–HFO coupling: a mechanism link to cognitive impairment

The analysis of the coupling between the IEDs and HFO in the hippocampus revealed a significant increase in the coupling strength between IEDs and HFO in the FAD + rats, particularly during later stage of AD pathology (the month 11 recording). As indicated by previous studies, IEDs and HFO could play a crucial role in the cognitive decline of AD animal models [3, 4]. IEDs have been shown to disrupt neuronal communication and induce neuroinflammation, thereby impairing synaptic function [87, 88]. HFOs, particularly in the hippocampus, have been observed to disturb memory processing and neural network dynamics [64, 89]. The coupling of IEDs with HFOs in the hippocampus that we see could reflect pathological network synchronization that undermines memory encoding and retrieval [89], leading to deficits in cognitive performance, as observed in our behavioral tests [30]. Given the increase in the occurrence of IEDs and HFOs in the hippocampus area of FAD + rats, coupling of IEDs and HFO may reflect pathological network synchronization that undermines memory encoding and retrieval, leading to deficits in cognitive performance.

FAD + rats showed cognitive decline in comparison to the FAD- rats. While both FAD + and FAD- groups showed deterioration in task performance over time, the FAD + rats displayed a more pronounced decline in their ability to recall learned information. This is a hallmark feature of AD-related memory deficits [90–93]. However, our findings are novel because we utilized a more complex and uniquely suited memory test, designed for higher cognition beyond that of mice, which has also been used in epilepsy models and not typically in AD models. The two-stage design of this memory test assess both the recall and learning function, and the separated two days letting a good period of sleep for memory consolidation [35, 94]. The results of regression analysis further highlighted the relationship between IED–HFO coupling strength and cognitive performance in the FAD + group. Specifically, a positive correlation was identified between the IED–HFO coupling and the time taken by animals to complete tasks. Furthermore, this correlation was not observed in the FAD- group, supporting the hypothesis that the pathological synchronization between IEDs and HFOs plays a critical role in the cognitive decline exclusively in animals with AD. Potentially, the pathological coupling of IEDs and HFOs disrupts the normal cognitive functions in hippocampal network dynamics. This disruption may manifest through impaired synaptic plasticity, neuroinflammation, and glial activation, which can in turn affect cognitive function [3, 30, 79, 95]. These mechanisms work together to exacerbate memory deficits and task performance, underscoring the critical role of abnormal brain activity in the pathophysiology of Alzheimer’s disease [96, 97].

### Limitations and future work

Like most previous research in this field, our animal model does not fully replicate the complexity of human AD. In addition, while we characterized region-specific IEDs and HFOs, our analysis focused exclusively on within-region coupling, particularly within the hippocampus. Brain network-level dysfunction is increasingly recognized as a key feature of AD-related cognitive decline. Future work should explore cross-regional IED–HFO dynamics, such as interactions between the hippocampus and prefrontal cortex to better understand how large-scale network hyperexcitability relates to memory impairment.

Another important direction is to evaluate whether anti-seizure medications can suppress IED–HFO coupling and improve cognitive outcomes in this model. For example, levetiracetam has been shown to reduce IEDs and enhance cognition in both mouse models [98] and human patients with AD [99]. Our recent work demonstrated that AD patients exhibited increased HFOs, detected by magnetoencephalography, and that levetiracetam treatment suppressed HFOs in patients with epileptic activity, coinciding with improved cognitive performance [65]. Assessing whether IED–HFO coupling provides a more sensitive indicator of clinically relevant network hyperexcitability than IEDs or HFOs alone will be a valuable avenue for future research. Collectively, these directions may clarify the mechanistic links between epileptiform activity and cognitive decline in AD.

## Conclusion

The present study, using an animal model of AD, suggests a potential mechanism through which IED–HFO coupling may contribute to cognitive decline and neuropathology in FAD + rats. This mechanism likely involves increased hippocampal IED rates leading to abnormal synchronization between neural circuits, thereby disrupting memory encoding, retrieval, and overall cognitive processing. These findings provide insight into how network-level hyperexcitability may underlie cognitive impairment in AD models and highlight IED–HFO coupling as a potential target for therapeutic interventions aimed at restoring normal neural activity and mitigating cognitive decline in AD.

## Supplementary Information

Below is the link to the electronic supplementary material.

Supplementary Material 1

## Data Availability

No datasets were generated or analysed during the current study.

## Abbreviations

AD: Alzheimer’s Disease
FAD +: Alzheimer’s Disease positive (transgenic rats expressing APP/PS1)
FAD-: Alzheimer’s Disease negative (non-transgenic littermates)
IEDs: Interictal epilepsy discharges
HFOs: High-frequency oscillations
pHFOs: Pathological high-frequency oscillations
SVD: Small vessel disease
LFP: Local field potential
EEG: Electroencephalogram
BSA: Bovine Serum Albumin
TBS: Tris-buffered saline
DAB: Diaminobenzidine
ABC: Avidin-biotin complex
WCBM: Water-cheese board maze
PETH: Peri-event time histogram
mPFC: Medial prefrontal cortex
SD: Standard deviation
NPY: Neuropeptide Y
