# Supplementary material for: Disruption of electrophysiological rhythms and memory impairment in an Alzheimer’s transgenic rat model
Source: Alzheimers Res Ther. 2025 Sep 1;17:200. doi: 10.1186/s13195-025-01841-4 (PMC12403914; doi:10.1186/s13195-025-01841-4)
Supplement: Supplementary file 1 — Supplementary Material 1 [file 13195_2025_1841_MOESM1_ESM.docx]

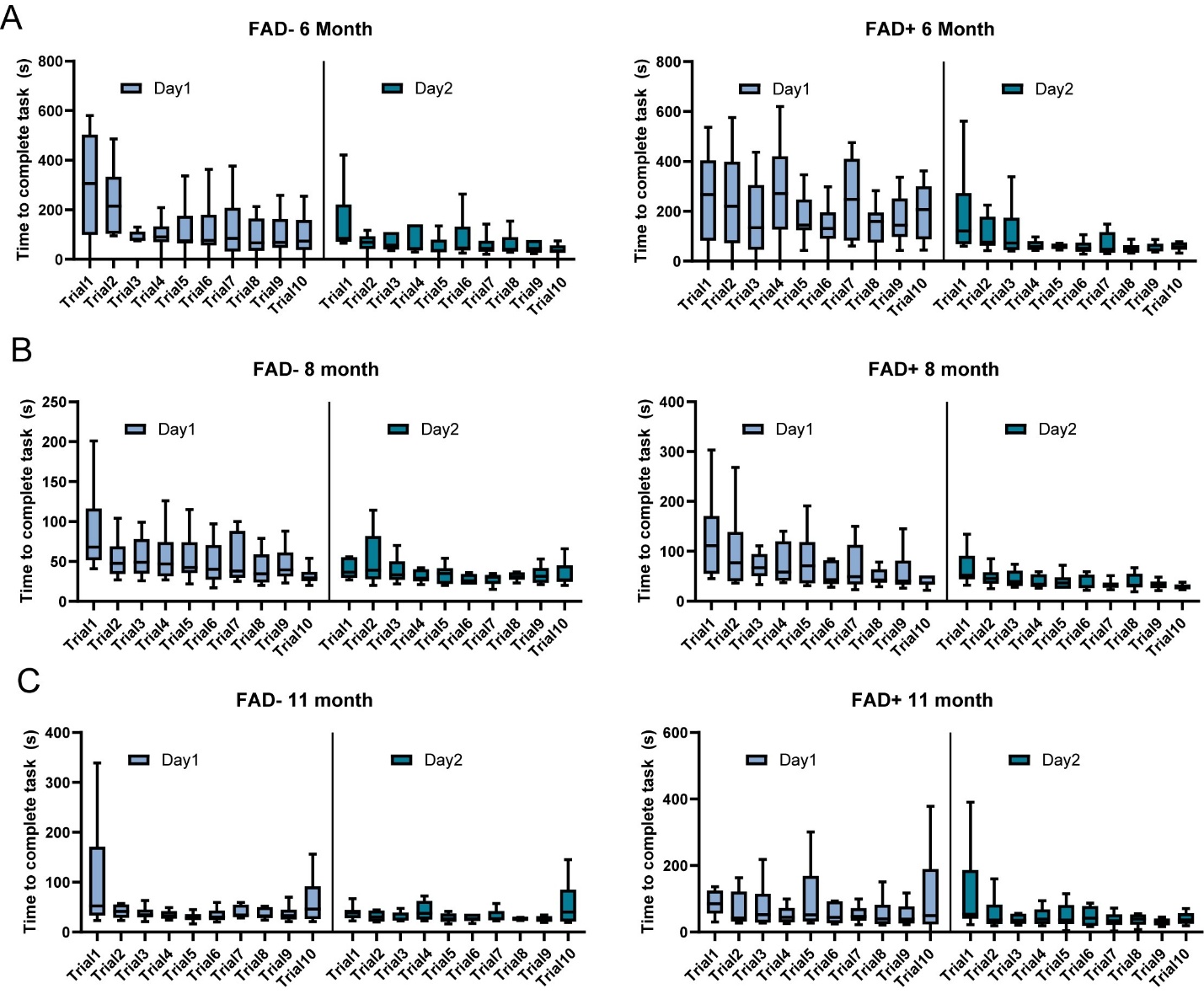


**Figure S1. Task completion times for each set of 10 trials in FAD− and FAD+ groups. A.** Six-month-old animals: total time to complete 10 trials for Learning (Day 1) and Recall (Day 2) phases, shown for FAD− and FAD+ groups. **B.** Eight-month-old animals: total time to complete 10 trials for Learning (Day 1) and Recall (Day 2) phases in FAD− and FAD+ groups. **C.** Eleven-month-old animals: total time to complete 10 trials for Learning (Day 1) and Recall (Day 2) phases in FAD− and FAD+ groups.


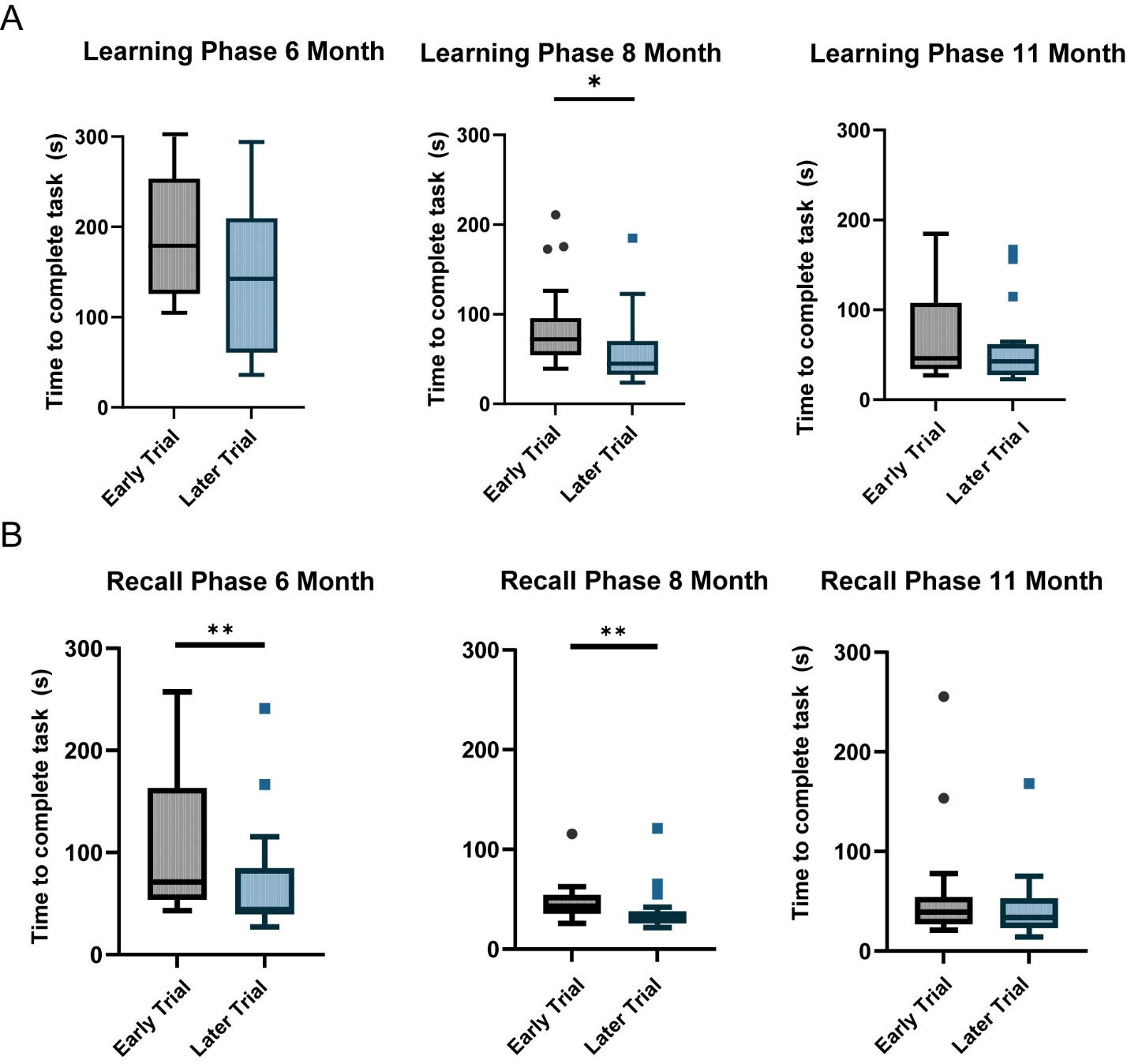


**Figure S2. Average total time to complete early (first 5 trials) versus later (last 5 trials) sets in FAD− and FAD+ groups. A.** Learning phase. Average total time to complete trials at 6 (p = 0.053), 8 (p = 0.037), and 11 (p = 0.431) months of age, comparing first 5 trials with last 5 trials. **B.** Recall phase. Average total time at 6 (p < 0.001), 8 (p = 0.008), and 11 (p = 0.055) months of age, comparing first 5 trials with last 5 trials.


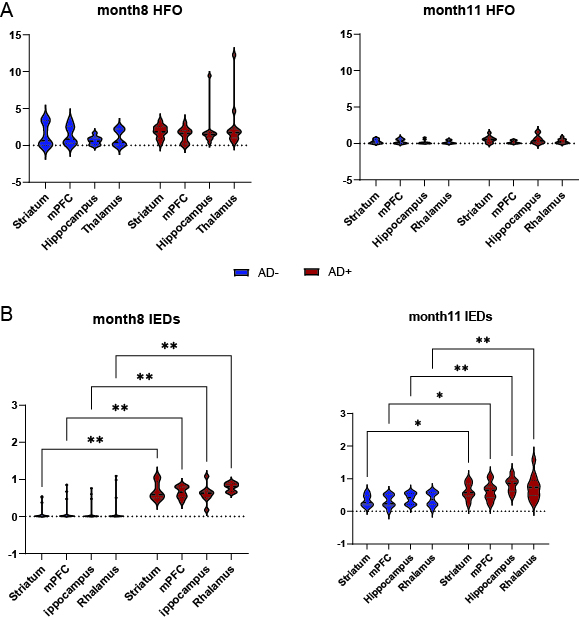


**Figure S3. Quantification of IED and HFO rates during REM sleep. A.** Average HFO rates at 8 and 11 months of age across four brain regions. **B.** Average IED rates at 8 and 11 months of age across four brain regions.


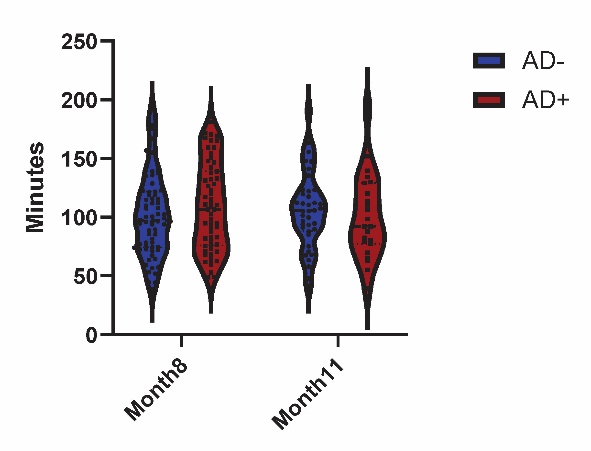


**Figure S4. Measurement of non-rapid eye movement (NREM) sleep at months 8 and 11 in the FAD- and FAD+ groups.**Totally we detected 333 hours of NREN EEG data. For each group, we measured their total EEG length as: 116, 71, 107, 39 hours for AD- (month 8&11) and AD+ (month 8&11), respectively. We randomly selected the EEG epoch length from each animal recording, with epoch length ranging from 28 to 192 minutes. There was no significant difference among individual EEG epoch length for the four groups (F(1,186) = 1.41, p = 0.24)).
